# 

**Published:** 1900-09-22

**Authors:** 


### NOTICE TO CORRESPONDENTS.

All MS., Letters, Books for Review, and other matters intended for the Editor should be addressed The Editor, "The Hospital," The Hospital Building, 28 & 29, Southampton Street, Strand, W.C.

The Editor cannot undertake to return rejected MS., even when accompanied by stamped directed envelope.

**Notice to Advertisers and Subscribers.**

All Advertisements, Orders for Copies of The Hospital, and Business Communications should be addressed to **THE MANAGER (Not to the Editor)**, The Hospital, The Hospital Building, 28 & 29, Southampton Street, Strand, London, W.C.

The Cost of Subscription, post free, is as follows:—Per week, 2½d.; per quarter, 2s. 8d.; per half-year, 5s. 3d.; per annum, 10s. 6d. Foreign:—Per annum, 15s. 2d., payable, in all cases, in advance.

**NOTICE.—Vol. XXVIII. of "The Hospital" (April to September, 1900), in handsome cloth binding, will shortly be on sale at the Office of this Journal, price 7s. 6d. Cases for binding the Numbers contained in this Volume 1s. 6d. Index to Vol. XXVIII., 2½d., post free.**

**The Monthly Part for October will be ready on October 1st. Price 6d., by post 8d.**

### Scraps and Gleanings.

The Scarborough Hospital Saturday Street Collection this year amounted to £276 odd.

The new fresh-air wing which has recently been opened at the Oriolet Hospital, Loughton, Essex, contains accommodation for four male and four female patients.

The Local Government Board have been petitioned by the Maidstone Town Council for sanction to borrow a sum of £3,252 for the purpose of carrying out extensions at the Maidstone Infectious Hospital. The inquiry by the Local Government Board into this application was held recently at Maidstone.

The new fever hospital of Ruchill, erected by the Corporation of Glasgow, was recently opened for the reception of patients. Its erection has occupied a period of six years, and the cost exceeds £250,000. The resident community, including medical and nursing staffs, and patients, averages 1,000 persons, the nursing staff alone numbering 200 members.

A sum of £3,700 is required by the committee of the Sheffield Royal Hospital before the end of 1900, in order to place them in a position to apply for the sum of £2,000 promised them by the Sheffield Town Trustees last November on condition that the amount required (£12,000) to complete the alterations and extensions was forthcoming by the end of this year. Up to the present only £6,389 has been received or promised.

The firm of contractors who have been entrusted with the erection of the new Royal Victoria Hospital, Belfast, have bound themselves to have the buildings erected within two years and three months from the date of the execution of the contract. The subscriptions received for the building fund amount to £81,000 odd, while the amount outstanding reaches almost £25,000. There is in the bank to the credit of the endowment fund a sum of £6,653.

A deputation from the Hull Royal Infirmary, headed by Sir James Reckitt, recently waited upon the Hull Board of Guardians with respect to the further extension of the infirmary and its maintenance. It has been estimated that the required extensions will involve an outlay of £50,000, and then an increase in the yearly expenditure of £4,000 would have to be faced. Sir James Reckitt proposed that to help to meet this the Guardians should levy a ½d. rate, to be given to the infirmary funds, which would bring them in about £900 per annum. The Guardians promised to consider the matter carefully. It was pointed out that the £900 which would accrue from the ½d. rate was only about half the actual annual cost to which the infirmary was put in treating pauper patients alone, which was now done by public subscription.

### The Student of Medicine.

### APPOINTMENTS.

T. W. L. Beales, L.R.C.P.Lond., M.R.C.S.Eng., appointed Medical Officer for the West Flegg District of the East and West Flegg Incorporation. A. B. Blacker, M.D.Durh., B.S., appointed Superintendent of the new X-Ray Department at the Hospital for Consumption, Brompton. D. R. P. Evans, L.R.C.P.Lond, M.R.C.S.Eng., L.S.A.Lond., appointed Medical Officer for the North Wimbledon District of the Kingston Union. G. Fowler, L.R.C.P., L.R.C.S.Edin., D.P.H.Eng., appointed Resident Assistant Medical Officer to the Sheffield City Hospitals. S. H. Hawley, M.B.Durh., appointed Medical Officer for the First District of the Walsall Union. W. C. Hirst, M.B., appointed Resident Assistant Medical Officer to the Leeds City Hospitals. H. W. Jackson, M.B.Lond., D.P.H., appointed Medical Officer for the Central District of the Middlesbrough Union. T. D. Kirk, M.D., M.Ch.R.U.I., appointed Medical Officer for the Forden District of the Forden Union. R. G. Knox, M.R.C.S.Eng., L.R.C.P.-Lond., appointed Certifying Factory Surgeon for the Freshford District of Somerset. E. M'Donald, M.B., B.Ch., appointed Medical Officer for the Nos. 1 and 2 Dispensary District of the Carlow Union. J. Marsh, M.B., C.M.Edin., appointed Medical Officer of Health for the Atherton Urban District. R. Morgan, M.D.R.U.I., appointed Certifying Factory Surgeon for the Bailyfeard District of the Kinsale Union, co. Cork. J. R. Prytherch, M.B., Ch.B.Edin., M.R.C.S.Eng., L.R.C.P.Lond., appointed Second Resident Medical Officer at Chester General Infirmary. F. W. Sydenham, M.D.Edin, D.P.H.Vict., appointed Medical Officer for the Second District of the Walsall Union. F. H. Waddy, M.D.Glasg., appointed Medical Officer for the South District of the Sheffield Union.

### NOW READY,

A REPRINT OF

### A NURSING HANDBOOK

TOGETHER WITH S. MAW, SON, AND THOMPSON'S

### COMPLETE CATALOGUE OF NURSING REQUISITES.

The above work is now republished at a cost of 1s. per copy. Messrs. S. M., S., and T. have been compelled to make this charge on account of the extreme popularity of the work and the great expense incurred in its production.

***Post Free on receipt of 1s.***

Nurses' Bags.

### S. MAW, SON, and THOMPSON,

7 to 12, ALDERSGATE STREET, LONDON, E.C.

